# Pembrolizumab Plus Gemcitabine in the Subset of Triple-Negative Advanced Breast Cancer Patients in the GEICAM/2015-04 (PANGEA-Breast) Study

**DOI:** 10.3390/cancers13215432

**Published:** 2021-10-29

**Authors:** Luis de la Cruz-Merino, María Gion, Josefina Cruz-Jurado, Vanesa Quiroga, Raquel Andrés, Fernando Moreno, Jose L. Alonso-Romero, Manuel Ramos, Esther Holgado, Javier Cortés, Elena López-Miranda, Fernando Henao-Carrasco, Natalia Palazón-Carrión, Luz M. Rodríguez, Isaac Ceballos, Maribel Casas, Sara Benito, Massimo Chiesa, Susana Bezares, Rosalia Caballero, Carlos Jiménez-Cortegana, Víctor Sánchez-Margalet, Federico Rojo

**Affiliations:** 1 Medical Oncology Department, Virgen Macarena University Hospital, Medicine Department University of Seville, 41009 Seville, Spain; ferheca@gmail.com (F.H.-C.); npalazoncarrion@gmail.com (N.P.-C.); 2 GEICAM Spanish Breast Cancer Group, 28703 San Sebastián de los Reyes, Spain; mariagion@gmail.com (M.G.); jcruzjurado@gmail.com (J.C.-J.); vquiroga@iconcologia.net (V.Q.); andresraquelc@gmail.com (R.A.); laventi2002@yahoo.es (F.M.); josel.alonso2@carm.es (J.L.A.-R.); manuel.ramos@cog.es (M.R.); eholgadomartin@gmail.com (E.H.); jacortes@vhio.net (J.C.); elemiranda@hotmail.com (E.L.-M.); luzmilva@gmail.com (L.M.R.); iceblen@gmail.com (I.C.); micasas@geicam.org (M.C.); sbenito@geicam.org (S.B.); mchiesa@geicam.org (M.C.); sbezares@geicam.org (S.B.); rcaballero@geicam.org (R.C.); frojo@fjd.es (F.R.); 3 Medical Oncology Department, Hospital Universitario Ramón y Cajal, 28034 Madrid, Spain; 4 Medical Oncology Department, Hospital Universitario de Canarias, 38320 Santa Cruz de Tenerife, Spain; 5 Medical Oncology Department, Badalona Applied Research Group in Oncology (B-ARGO Group), Catalan Institute of Oncology, 08908 Badalona, Spain; 6 Medical Oncology Department, Hospital Clínico Universitario Lozano Blesa, 50009 Zaragoza, Spain; 7 Medical Oncology Department, Hospital Clínico Universitario San Carlos, 28040 Madrid, Spain; 8 Medical Oncology Department, Hospital Clínico Universitario Virgen de la Arrixaca, Instituto Murciano de Investigación Biomédica (IMIB), 30120 Murcia, Spain; 9 Medical Oncology Department, Centro Oncológico de Galicia, 15009 A Coruña, Spain; 10 Medical Oncology Department, Hospital La Luz (Quironsalud Group), 28003 Madrid, Spain; 11 International Breast Cancer Center (IBCC), Quirón Teknon Hospital (Quironsalud Group), 08017 Barcelona, Spain; 12 Vall d’Hebron Institute of Oncology (VHIO), 08035 Barcelona, Spain; 13 Department of Medicine, Faculty of Biomedical and Health Sciences, Universidad Europea de Madrid, 28670 Madrid, Spain; 14 Medical Biochemistry and Molecular Biology and Immunology Department, Virgen Macarena University Hospital, University of Seville, 41009 Seville, Spain; cjcortegana@gmail.com (C.J.-C.); margalet@us.es (V.S.-M.); 15 Hospital Universitario Fundación Jiménez Díaz, 28040 Madrid, Spain; 16 Centro de Investigación Biomédica en Red de Oncología (CIBERONC), Instituto de Salud Carlos III (ISCIII), 28029 Madrid, Spain

**Keywords:** phase II, triple-negative, breast cancer, pembrolizumab, immunotherapy, biomarkers, PD-L1, TILs, MDSCs

## Abstract

**Simple Summary:**

Advanced triple-negative breast cancer (TNBC) remains an extremely challenging situation in oncology, where new therapeutical strategies are desperately needed. Immunotherapy has opened a window of opportunity in this setting, with some promising results with chemo-immunotherapeutic schedules based on anti-PD1/PD-L1 agents, especially in the PD-L1-positive cohort. However, there is certainly room for improvement; thus, new schemes that could potentially boost synergism against cancer cells must be explored. This work analyzes the effects of combination therapy with anti-PD1 (pembrolizumab) and gemcitabine, specifically in the TNBC cohort of the PANGEA-Breast trial. Patients included in this study were not selected by PD-L1 status, and most of them were also heavily pretreated, which could explain the modest objective response rate of 15% achieved. Complementary translational subanalyses, focused on T infiltrating lymphocytes, myeloid-derived suppressor cells, and PD-L1 were accomplished.

**Abstract:**

The PANGEA-Breast trial evaluated a new chemo-immunotherapeutic combination that would synergistically induce long-term clinical benefit in HER2-negative advanced breast cancer patients. Treatment consisted of 21-day cycles of 200 mg of pembrolizumab (day 1) plus gemcitabine (days 1 and 8). The primary objective was the objective response rate (ORR). The tumor infiltrating lymphocytes (TILs) density and PD-L1 expression in tumor, and the myeloid-derived suppressor cells (MDSCs) level in peripheral blood, were analyzed to explore associations with treatment efficacy. Considering a two-stage Simon’s design, the study recruitment was stopped after its first stage as statistical assumptions were not met. A subset of 21 triple-negative breast cancer (TNBC) patients was enrolled. Their median age was 49 years; 15 patients had visceral involvement, and 16 had ≤3 metastatic locations. Treatment discontinuation due to progressive disease (PD) was reported in 16 patients. ORR was 15% (95% CI 3.2–37.9). Four patients were on treatment >6 months before PD. Grade ≥3 treatment-related adverse events were observed in 8 patients, where neutropenia was the most common. No association was found between TILs density, PD-L1 expression or MDSCs levels and treatment efficacy. ORR in TNBC patients also did not meet the assumptions, but 20% were on treatment >6 months.

## 1. Introduction

Breast cancer (BC) represents the leading cause of cancer deaths in women in Western countries. Although great advances have been achieved in recent decades, when the disease spreads to distant sites, most patients ultimately die due to disease progression. In addition, BC is recognized as a heterogeneous disease, where the triple-negative breast cancer (TNBC) subgroup has a poorer prognosis and more aggressive behavior. Although systemic targeted therapies are still largely unaffordable in TNBC patients, immunotherapy has recently been introduced in this scenario due to the higher levels of potential immune biomarkers in its microenvironment, such as higher densities of tumor-infiltrating lymphocytes (TILs) and PD-L1 positivity. Early results with chemo-immunotherapeutic schedules in phase I–II trials showed some signals of activity [1,2], especially in enriched (PD-L1 and TILs) populations, and therefore, a set of phase III clinical trials were initiated. At this time, results are contradictory, since two of these phase III clinical trials (IMpassion-130 and Keynote-355, with atezolizumab and pembrolizumab, respectively) in the first-line setting have communicated positive results in the PD-L1-positive TNBC cohort. However, the IMpassion-131 trial, also in first-line and with a similar design, recently reported negative outcomes in their primary and secondary endpoints. Other studies—such as the IMpassion-132 trial in inoperable locally advanced/metastatic TNBC recurring ≤12 months after completing standard (neo)adjuvant chemotherapy [3], with atezolizumab and capecitabine or carboplatin plus gemcitabine—will help to clarify the real value of modern immunotherapy in this disease. Moreover, translational data that could definitively establish reliable biomarkers are certainly needed, as patients benefiting from immunotherapy should be better characterized. Lately, to summarize the current knowledge regarding immunotherapy in BC, the Society for Immunotherapy of Cancer (SITC) has published a practice guideline that revises the available data and makes some proposals and recommendations [4].

In the PANGEA-Breast trial, we aimed to test if a novel chemo-immunotherapy combination with gemcitabine and pembrolizumab could be feasible and obtain meaningful clinical and long-term responses in pretreated advanced BC (ABC) patients, with the TNBC or luminal A/B subtype according to St. Gallen subtype classification [5] irrespective of their PD-L1 status. Gemcitabine was chosen to be combined with the anti-PD-L1 monoclonal antibody pembrolizumab, as some immunogenic properties through the elimination of myeloid-derived suppressor cells (MDSCs) and lymphocytes T regulators (Tregs) in preclinical tumor models were previously suggested [6,7,8,9].

## 2. Results

### 2.1. Patients’ Characteristics

From June 2017 to May 2018, twenty-one TNBC patients were recruited in eight Spanish sites, representing 58% of the study population. A Simon’s minimax two-stage design was used for this study; recruitment was stopped at its first stage as the statistical assumptions or the primary objective to continue to the second stage were not met.

The characteristics of this subset of patients are described Table 1 with similarities with the whole study population regarding the distribution of different characteristics. The median age was 49 years, most patients were postmenopausal, and 52% had an Eastern Cooperative Oncology Group performance status (ECOG PS) scale value of 0. In addition, a high histological grade and a Ki67 index level of at least 20% were reported in more than 50% of these TNBC patients.

Almost 80% of patients had up to three metastatic locations, and around 70% of patients had visceral involvement, mainly in the liver (57%). Most of the patients were diagnosed with early BC, and the median time between the first BC diagnosis and the inclusion in the study was four years. Regarding the previous anticancer therapy for advanced disease, TNBC patients had a median of 2 prior lines (range 0–8); 19 patients were previously treated with chemotherapy, with gemcitabine administered in only 4 patients, who represent less than 20% of this subpopulation. In addition, four patients received endocrine therapy, and five received other drugs for hormone-receptor-positive or HER2-positive breast cancers.

### 2.2. Treatment Exposure

This subpopulation of TNBC patients received up to 24 cycles with a median of 3 cycles for both pembrolizumab and gemcitabine. The median relative dose intensity of the combination at the recommended dose for phase II (RP2D) was 100% (100%–100%) and 78% (39%–100%) for pembrolizumab and gemcitabine, respectively.

The RP2D was the dose level 0, consisting of pembrolizumab at 200 mg on day 1 and gemcitabine at 1250 mg/m^2^ on days 1 and 8, for cycles of 21 days (Supplementary Figure S1).

Six (29%) patients experienced any delays of pembrolizumab administration, due to adverse events in 4 (19%) patients; these adverse events included liver function tests alterations, pyrexia, arthralgia, and infections. Eighteen (86%) patients observed dose modifications for gemcitabine, with omissions being the most frequent modification followed by reductions and then delays; adverse events were the most common reason for all types of modifications.

The main reasons for treatment discontinuation included progressive disease (PD) in 16 (76%) patients, death in 2 (10%), and adverse event (grade 3 respiratory failure) and physician’s decision in 1 (5%) patient each. At the time of this analysis, one patient was still on treatment.

### 2.3. Efficacy

**Figure 1:**
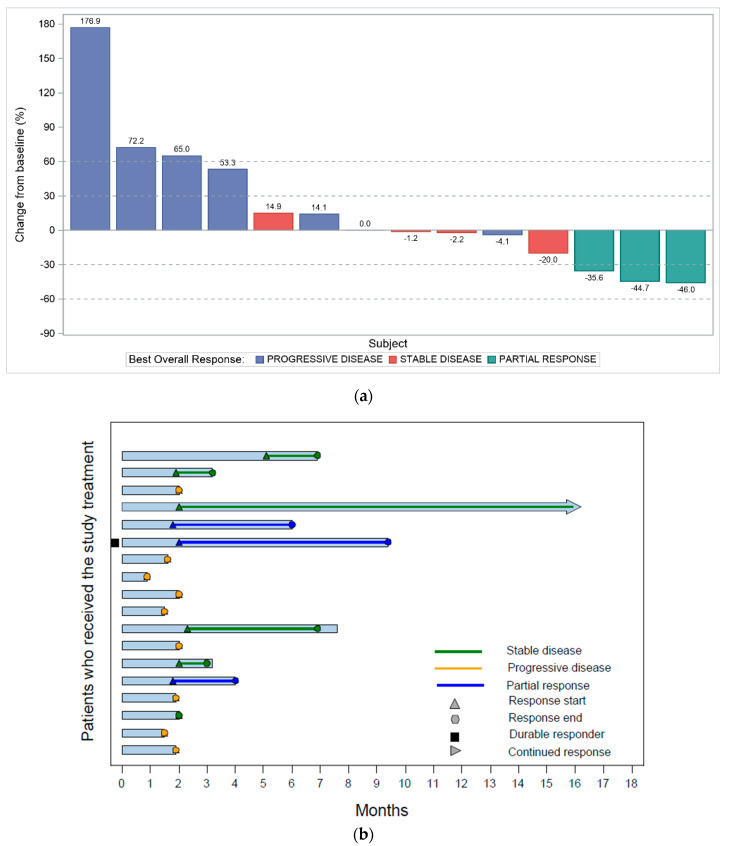
The best overall tumor response per each individual TNBC patient and its duration. (**a**) Waterfall plot. The waterfall plot presents each individual patient’s response to the study treatment based on the change of the sum of the target lesions diameters. The vertical bars are drawn from the baseline, either in the positive or negative direction to depict the change from baseline. Fourteen of the twenty patients analyzed as part of the efficacy population are represented (three patients did not have any tumor response after the beginning of the study treatment as explained in the efficacy section, and the other three patients did not have any change to be represented: two patients progressed with the development of new lesions and another patient had clinical PD). (**b**) Swimmer plot. The swimmer plot shows multiple pieces of each individual patient’s response; the graph includes a bar showing the length of treatment duration and indicators for the start (triangle) and end (circle) of each response episode, classified by a partial response (blue line), stable disease (green line) or progressive disease (dark yellow line), as well as an indicator showing whether the patient is a durable responder (black square) and whether the patient is still on the corresponding response (lying down triangle). In this graphic, the twenty-one TNBC patients are considered as the intention-to-treat population, however, the three patients without tumor response assessment after the beginning of the study treatment are not included.

According to the study protocol and the statistical analysis plan, the objective response rate (ORR) was calculated considering the efficacy population, a subset of the intention-to-treat (ITT) population with measurable disease, who had received at least one dose of study treatment and had at least one tumor response assessment according to RECIST version 1.1 (unless progressive disease (PD), death or unacceptable toxicity was seen before the first tumor response assessment was performed) and without certain major protocol deviations detailed in the protocol deviations manual. One patient was excluded from the efficacy population because of a major protocol deviation, and three patients did not have a tumor response assessment after the beginning of the study treatment because of PD, death secondary to breast cancer and unacceptable toxicity. Twenty patients were considered for the analysis. The ORR was 15% (*n* = 3; 95% confidence interval [CI] 3–38), with partial responses (PR) only. Five (25%) patients achieved stable disease (SD) (in one case, this lasted more than 6 months), and nine (45%) patients experienced PD. The clinical benefit rate, including stable diseases of any duration, was 40% (*n* = 8; 95% CI 19–64). The median duration of response was of 4 months (95% CI 2–7), the median progression-free survival (PFS) was 2 months (95% CI 2–3), and the median overall survival (OS) was 8 months (95% CI 3–10). Four patients were on treatment more than 6 months at the cut-off date for the current analysis. The best overall tumor responses per patient and the duration of responses are represented in Figure 1. The efficacy based on immune-related (ir) response criteria, showed similar results.

### 2.4. Safety

Almost all patients (20 [95%]) experienced an adverse event, and adverse events related to the study treatment occurred in 14 (67%) patients. Adverse events of grade 3 were reported in nine (43%) patients and of grade 4 in four (19%). Of these adverse events, the relationship with the study treatment was established by the investigators in eight (38%) patients. Serious adverse events were reported in nine (43%) patients, and adverse events related to treatment in two cases. No grade 5 adverse events related to the treatment were reported.

Table 2 lists the treatment-emergent adverse events irrespective of the causal relationship with the study treatment and with a frequency of at least 5% in any grade of severity.

### 2.5. Association between TILs Density and PD-L1 Expression with Treatment Efficacy

Higher ORR was observed in patients showing higher TILs infiltration (≥20%) and in patients with higher PD-L1 levels (Combined Positive Score [CPS] ≥10, ≥20) (Table 3). Nevertheless, no significant associations were observed between distribution according to different cut-offs of TILs density or PD-L1 expression (Immune Cell [IC] score/CPS) and either OR or PFS (Table 3). Analysis of the distribution of median TILs density according to tumor response shows that patients achieving a complete or partial response tend to present higher levels of infiltration by lymphocytes (Supplementary Figure S2A).

Nine (53%) patients were PD-L1 positive, considering either IC ≥ 1% or CPS ≥ 1 (seven of them were positive for both scores). Median PD-L1 CPS values were higher in patients achieving an objective tumor response (OR) (complete or partial) (Supplementary Figure S2B). PD-L1 positive IC scores seemed to be more frequent in patients with no OR (Supplementary Figure S3).

The combination of TILs and PD-L1 (CPS) cut-offs allowed us to observe that patients with both higher TILs infiltration (≥20%) and higher CPS (≥10) showed better ORR, whereas patients with lower indexes (TILs < 5% and CPS < 10) presented an opposite tendency (Table 3).

Finally, a significant positive correlation between TILs levels and PD-L1 expression was observed (correlation coefficient = 0.543; *p* = 0.002967) (Supplementary Figure S4).

### 2.6. Association between Myeloid-Derived Stem Cells (MDSC) Levels and Efficacy

**Figure 2:**
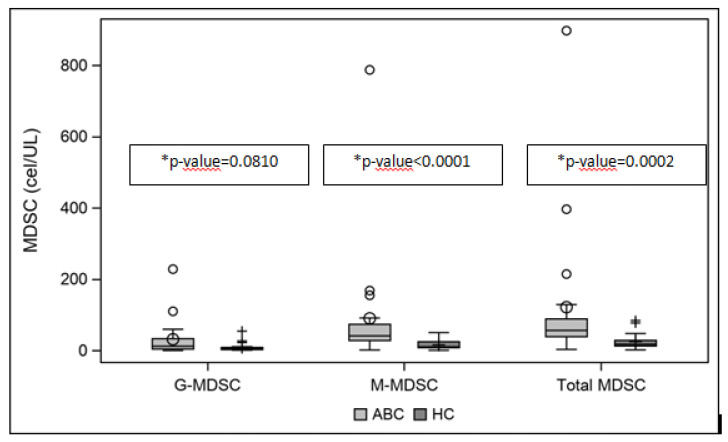
Baseline median values of MDSCs (G-, M-, total; cells/µL) comparison between ABC TN patients and healthy cohort. * Wilcoxon–Mann–Whitney test. Abbreviations: MDSCs = myeloid derived suppressor cells; M-MDSCs = monocytic MDSCs; G-MDSCs = granulocytic MDSCs; ABC = advance breast cancer; TN= triple-negative; HC = healthy cohort.

Study patients showed significantly higher concentrations of total MDSCs at baseline than a healthy control cohort (median values of 57 vs. 18 cells/µL, respectively, *p* = 0.0002). This difference was especially marked in M-MDSCs (median values of 41 vs. 10 cells/µL, *p* < 0.0001) rather than G-MDSCs (median values of 12 vs. 5 cells/µL, *p* = 0.0810) (Figure 2).

Analysis of the variation of MDSCs values along treatment (baseline-cycle 3 and baseline-cycle 6/end of treatment [EOT]) revealed no significant differences associated with clinical benefit (CB) (Table 4). In the same way, no significant differences in MDSCs levels between patients showing CB and patients showing PD could be observed at any study time point (Table 4).

## 3. Discussion

In the TNBC cohort of the PANGEA-Breast trial, the chemo-immunotherapy combination of gemcitabine and pembrolizumab achieved a modest ORR of 15.2%, and no long-term responders were observed, although four patients were on treatment more than 6 months. However, these data confirm that patients benefiting from chemo-immunotherapy schedules in BC must be carefully chosen. At this time, only unresectable TNBC PD-L1 positive patients in the first-line setting seem to be potential candidates for therapeutical strategies integrating anti-PD1/PD-L1 monoclonal antibodies, as the IMpassion-130 and Keynote-355 trials, with atezolizumab and pembrolizumab, respectively, revealed. Of note, pembrolizumab was recently approved by the FDA for high-risk, early stage TNBC based on the KEYNOTE-522 trial [10].

Nevertheless, this work yields some interesting findings. In the translational analyses, it was shown that patients with higher TILs or PD-L1 (TILs ≥ 20% and CPS ≥ 20) achieved better ORR. These results are concordant with those of the Keynote-119 trial [11], in which pretreated metastatic TNBC patients were randomized to pembrolizumab vs. the investigator´s choice (capecitabine, eribulin, vinorelbine or gemcitabine). 

Beyond TILs and PD-L1 analysis in tissue, data obtained with respect to MDSCs in peripheral blood in this trial were intriguing. MDSCs are immature myeloid cells expanded during pathological conditions with pleiotropic immunosuppressive properties [6,12,13]. MDSCs role as therapeutic target in cancer seems progressively clearer, and therefore, different strategies to deplete and alter their function or induce their differentiation are being explored [14].

In our work, MDSCs levels in the peripheral blood of advanced TNBC patients were increased over the healthy donors that served as controls. Therefore, it can be inferred that tumors induce an immunosuppressive status that can be easily measured using blood samples in the patients. These findings in BC were previously suggested by our group and other researchers [15,16,17,18]. In an attempt to deplete MDSCs levels and improve clinical outcomes by boosting the immune response, gemcitabine was chosen as a partner of pembrolizumab in this work [7]. Unfortunately, this combination failed to obtain meaningful clinical outcomes, however, MDSCs levels decreased numerically in patients with CB vs. PD, with differences being particularly evident at cycle 3 (12.9 vs. 60.7 cells/µL). Undoubtedly, additional studies with a larger sample are needed to clarify the potential role of MDSCs as a therapeutic target in BC.

This study has some limitations that are worth noting. Firstly, the sample size is scarce (*n* = 21) and represents a subset of the phase II non-randomized PANGEA-Breast study that also included luminal A/B patients. Furthermore, the patient population was unselected and, therefore, not enriched by PD-L1 and/or TILs and, last but not least, women included represented a heavily pretreated population (≥3 lines in 43% of patients), which could explain the low efficacy rates observed. Nevertheless, results obtained in this advanced TNBC cohort are concordant with those obtained in previous studies, reinforcing the current evidence that postulates a benefit for immunotherapy based on immune-checkpoint inhibitors strictly in the first-line setting, and in enriched populations (PD-L1 positive by IC SP-142 and/or CPS).

## 4. Materials and Methods

### 4.1. Study Design

The PANGEA-Breast was an open-label, single-arm, multicenter phase II trial conducted in nine sites in Spain. Pembrolizumab in combination with gemcitabine was evaluated in patients with HER2-negative advanced breast cancer (ABC), including triple-negative (TN) and hormone receptor (HR)-positive disease, with a balanced distribution between both cohorts. In this manuscript, we present the data from the TN subpopulation.

There was an initial exploratory run-in-phase, in which the Recommended Phase II Dose (RP2D) was defined [19] Eligible patients were enrolled and treated with pembrolizumab plus gemcitabine. The primary objective was to evaluate the efficacy of the combination in terms of objective response rate (ORR) defined as complete response (CR) plus partial response (PR) according to the Response Evaluation Criteria in Solid Tumors (RECIST) version 1.1. Secondary objectives were progression-free survival (PFS), defined as the time from enrolment to documented progressive disease (PD) or death from any cause; clinical benefit rate (CBR), defined as CR plus PR plus stable disease (SD) for at least 24 weeks; duration of response (DoR), defined as the time from the first documentation of objective tumor response (CR or PR) until the date of first observation of PD; OS, defined as the time from the date of enrolment to the date of death from any cause; and safety and tolerability of the combination assessed according to National Cancer Institute Common Terminology Criteria for Adverse Events (NCI CTCAE) version 4.0. The exploratory objectives were to assess the efficacy based on immune-related (ir) response criteria; search for tumor tissue and peripheral blood biomarkers of clinical activity, analyzing and correlating a set of immune biomarkers with the evolution of the disease and efficacy of the combination (ORR, CBR, DoR and PFS) paying special attention to long-term responders; and compare this set of biomarkers data from cohorts of healthy volunteers with data from patients included in the study.

The PANGEA-Breast study, from which the subpopulation of TNBC patients is considered for the current analysis, was conducted in accordance with the International Conference on Harmonization Good Clinical Practice Guidelines (ICH GCP) and the Declaration of Helsinki, approved by the institutions’ ethical review boards of the participating sites and health authorities in Spain, and registered at ClinicalTrials.gov and EudraCT (identifiers: NCT03025880 and 2016-001779-54, respectively).

### 4.2. Patients

Key inclusion criteria included: women aged 18 years or older; HER2-negative ABC by IHC and/or in situ hybridization (ISH) based on local testing on the most recent tumor biopsy following current guidelines for biomarker interpretation; ≥1 measurable lesion per RECIST 1.1; an Eastern Cooperative Oncology Group performance status (ECOG PS) of 0 or 1; prior treatment with anthracyclines and taxanes (unless clinically contraindicated); ≥2 prior lines of endocrine therapy and ≤4 prior chemotherapy (CT) lines for ABC; patient agreement to the collection of a fresh metastatic tumor biopsy at the time of inclusion and at PD; adequate organ function; adequate contraception and a negative pregnancy test for women of child-bearing potential. Key exclusion criteria included: previous therapy with anti-PD-1/anti-PD-L1/anti-PD-L2 agents; prior anticancer monoclonal antibody within 4 weeks of pembrolizumab dose; prior CT, targeted small molecule therapy, or radiation therapy within 2 weeks of pembrolizumab dose; active brain metastases (treated and stable without steroids were allowed); having received a live vaccine within 30 days of planned start of study therapy; an active autoimmune disease, or active infection, or active *Bacillus Tuberculosis* or Human Immunodeficiency Virus or active Hepatitis B or C; use of systemic steroid therapy or other immunosuppressive therapy within 7 days of pembrolizumab dose.

Healthy controls from the University Hospital Virgen Macarena in Seville gave written informed consent to participate in the analysis.

### 4.3. Treatment Plan

Patients received 200 mg pembrolizumab IV on day 1, and 1250 mg/m^2^ gemcitabine IV on day 1 and 8, of each 21-day cycle until objective PD, clinical PD (under the investigator’s criterion), unacceptable toxicity, death, or withdrawal of consent, whichever occurred first. Patients discontinuing the study treatment entered the follow-up phase.

### 4.4. Efficacy and Toxicity Evaluation Procedures

Baseline assessments were performed within 28 days from the start of study treatment. These included tumor assessment by radiological tests accepted by RECIST v1.1, standard 12-lead electrocardiogram, hematology, biochemistry, coagulation test (international normalized ratio/prothrombin time and activated partial thromboplastin time), thyroid hormones (triiodothyronine (T3) or free T3 (FT3), free thyroxine and thyroid stimulating hormone), urinalysis, pregnancy test, physical examination (including vital signs) and ECOG PS evaluation. Tumor assessments were performed every 9 weeks until PD, using the same measurement method as at baseline. Adverse events were graded using the NCI CTCAE version 4.0. Other safety endpoints included regular monitoring of vital signs, hematology, biochemistry, coagulation test, thyroid hormones, and urinalysis.

### 4.5. TILs and PD-L1 Assessments

Seventeen available pretreatment metastatic tumor samples were assessed for TILs density defined as the percentage of occupied stromal area upon hematoxylin and eosin (H&E) staining of formalin-fixed paraffin-embedded tissue slides according to suggested international guidelines [20]. Cut-offs explored for TILs evaluation were ≥5%, ≥10%, and ≥20%.

PD-L1 IHC expression was assessed in 16 pretreatment metastatic tumor samples using monoclonal anti-PD-L1 antibody clone 22C3 (Merck). The ICs (Immune Cells) score (percentage of tumor area occupied by positive lymphocytes, macrophages, dendritic cells, and granulocytes identified by H&E staining) and CPS (PD-L1 stained cells (tumor cells, lymphocytes, and macrophages) number divided by the total number of viable tumor cells, multiplied by 100) were obtained. PD-L1 scores were considered positive if ≥1. Cut-offs (≥5, ≥10, and ≥20) were additionally explored for CPS.

Logistic and Cox regression models were used to evaluate the association between TILs density and PD-L1 expression with treatment efficacy in terms of OR, CB (CR + PR + SD ≥24 weeks) and PFS, according to RECIST 1.1. Wilcoxon signed-rank test was used to compare MDSC values between baseline and cycle 3 (C3), and baseline and cycle 6 or at the end of treatment, whichever occurred first (C6/EOT). Additionally, the Wilcoxon–Mann–Whitney test was used to compare MDSC values between CB and PD in baseline, C3 and C6/EOT and to compare MDSC values between ABC and HC cohorts.

### 4.6. Staining Immunophenotyping of Whole Peripheral Blood by Flow Cytometry Analysis

Blood samples were collected in EDTA-K3 tubes at baseline, before C3 and C6/EOT, whichever occurred first, to determine MDSCs counts. Cell populations were determined by flow cytometry of whole blood using the BD FACSCanto™ system (BD Bioscience, Franklin Lakes, NJ, USA). M-MDSCs were determined as CD45+ CD11b+ CD33+ HLA-DR- CD14+ CD15- and G-MDSC as CD45+ CD11b +CD33+ HLA-DR- CD14- CD15+. Monoclonal antibodies: antibodies were obtained from Becton Dickinson Immunocytometry Systems (BDIS, San Jose, CA, USA) and were used at the manufacturer’s recommended concentrations. Antibodies included: PerCP-Cy5.5 Mouse Anti-Human CD45 (Catalog no. 564105), APC-Cy7 Rat Anti-CD11b (Catalog no. 557657), PE Mouse Anti-Human CD33 (Catalog no. 555450), PE-Cy7 Mouse Anti-Human HLA-DR (Catalog no. 560651), FITC Mouse Anti-Human CD14 (Catalog no. 555397) and APC Mouse Anti-Human CD15 (Catalog no. 551376).

### 4.7. Statistical Analysis

A Simon’s minimax two-stage design was employed for the phase II part of the study with the possibility of stopping early due to lack of response. The sample size was calculated testing the null hypothesis that gemcitabine produced an ORR of around 20%. With the study combination, the alternative hypothesis was that the ORR was 35% (an absolute increase of 15%); with an alpha error of 0.05 and a statistical power of 80%, 53 evaluable patients were required. The first stage should include 31 evaluable patients, and if at least 7 presented a response, recruitment would continue to include 53 evaluable patients. The null hypothesis of 20% should be rejected if 16 or more responses were observed in 53 patients.

The SAS Enterprise Guide (version 7.1) was used for all analyses.

## 5. Conclusions

This work focused on the TNBC subgroup of the PANGEA-Breast trial treated with a combo of pembrolizumab and gemcitabine. The low response rate achieved (15.2%) discarded this schedule for future investigations, at least in unselected populations, not enriched by PD-L1 and/or TILs. For future clinical trials in this setting, a better selection of advanced TNBC patients would be advisable. At this point, a combined analysis of TILs and PD-L1 expression could refine the cohort of patients suitable for immunotherapy. In addition, different therapeutic approaches aiming to induce a wider and more effective antitumoral immune stimulation are certainly needed, as current results of chemo-immunotherapy schedules in ABC seem globally modest with respect to those seen in other tumor types.

## Figures and Tables

**Table 1:** Patients’ and disease characteristics of the triple-negative breast cancer subpopulation.

Characteristics of the TNBC Patients	*n* = 21
**Age, years [median (range)]**	49 (31–74)
<65, *n* (%)	20 (95)
≥65, *n* (%)	1 (5)
**Menopausal status, *n* (%)**	
Postmenopausal	15 (71)
Premenopausal	6 (29)
**ECOG Performance Status, *n* (%)**	
0	11 (52)
1	10 (48)
**Time between the first BC diagnosis and the study inclusion, years [median (range)]**	4 (1–11)
**Number of metastatic locations, *n* (%)**	
1	7 (33)
2	4 (19)
3	5 (24)
4	4 (19)
5	1 (5)
**Metastatic locations, *n* (%)**	
Visceral	15 (71)
Non-visceral only	6 (29)
Organs most frequently involved:	
Liver	12 (57)
Bone	10 (48)
Lymph nodes	10 (48)
Lung	6 (29)
Skin	3 (14)
Pleural cavity	2 (10)
Soft tissue	2 (10)
**Histological type, *n* (%)**	
Invasive ductal carcinoma	19 (91)
Invasive lobular carcinoma	1 (5)
Invasive squamous carcinoma	1 (5)
**Histological grade, *n* (%)**	
2	6 (29)
3	11 (52)
Unknown	4 (19)
**Ki67 expression in % (local laboratory)**	
Median (range)	43 (20–95)
≥20%, *n* (%)	12 (57)
Unknown, *n* (%)	9 (43)
**Prior therapies for early disease, *n* (%)**	*n* = 19
Chemotherapy [(neo)adjuvant]	19 (91)
Endocrine therapy (adjuvant)	9 (43)
Anti-HER2 therapy [(neo)adjuvant]	2 (10)
**Number of prior lines for advanced disease, *n* (%)**	
Median (range)	2 (0–8)
0	1 (5)
1	9 (43)
2	2 (10)
≥3	9 (43)
**Most frequent prior therapies for advanced disease, *n* (%)**	
Chemotherapy	19 (91)
Taxanes or capecitabine	13 (62)
Anthracyclines, carboplatin, or eribulin	6 (29)
Gemcitabine	4 (19)
Other (vinorelbine, etoposide, etc.)	7 (34)
Endocrine therapy (AI, fulvestrant, and/or tamoxifen)	4 (19)
Bevacizumab	5 (24)
AntiHER2 therapies	2 (10)
mTOR inhibitor	2 (9.5)
Other (CDK4/6 inhibitor, PARP inhibitor, or lurbinectedin)	3 (15)

Abbreviations: TNBC: triple-negative breast cancer; *n*: number of patients; ECOG: Eastern Cooperative Oncology Group; AI: aromatase inhibitor; mTOR: mechanistic target of rapamycin; CDK: cyclin-dependent kinase; PARP: poly (ADP-ribose) polymerase.

**Table 2:** Treatment-emergent adverse events by grade according to NCI-CTCAE (version 4.03) and frequency of at least 5% in any grade.

Safety Population (*n* = 21)				
Adverse Event Term	Grade 1, *n* (%)	Grade 2, *n* (%)	Grade 3, *n* (%)	Grade 4, *n* (%)
Patients with any TEAE	14 (67)	16 (76)	10 (48)	4 (19)
Pyrexia	7 (33)	1 (5)	0	0
Asthenia	4 (19)	6 (29)	2 (10)	0
Anemia	3 (14)	2 (10)	2 (10)	0
Nausea	4 (19)	2 (10)	0	0
Decreased appetite	3 (14)	1 (5)	0	0
Musculoskeletal chest pain	2 (10)	0	0	0
Upper respiratory tract infection	2 (10)	0	0	0
Arthralgia	0	2 (10)	0	0
Abdominal pain upper	0	2 (10)	0	0
Constipation	1 (5)	2 (10)	1 (5)	0
Pain of skin	0	2 (10)	0	0
Tachycardia	0	2 (10)	0	0
Hypertension	0	2 (10)	0	0
Neutrophil count decreased	0	5 (24)	4 (19)	2 (10)

Abbreviations: TEAE = treatment-emergent adverse events; NCI-CTCAE = National Cancer Institute Common Terminology Criteria for Adverse Events; *n*: number of patients.

**Table 3:** ORR and PFS according to different TILs, PD-L1 and combined TILs/PD-L1 cut-offs. Logistic and Cox regression models have been used to analyze the association between TILs density and PD-L1 expression (IC and CPS scores) with OR (CR + PR) and PFS. Median PFS were calculated by Kaplan–Meier.

TILs and PD-L1 Score Levels		ORR (%) *p*-Value Odds Ratio (95% CI)	Median PFS (95% CI) *p*-Value Hazard Ratio (95% CI)
TILs (*n* = 17) Cut-off (*n*, %)	**≥5%** (13, 76) vs. **<5%** (4, 24)	15.3% vs. 0% NA	2.6 (1.3, 6.1) vs. 2.0 (1.9, NE) 0.536 1.50 (0.41, 5.45)
	**≥10%** (9, 57) vs. **<10%** (8, 43)	11.1% vs. 12.5% 0.929 0.87 (0.05, 16.74)	3.1 (1.2, 7.1) vs. 2.0 (1.5, 6.1) 0.763 0.85 (0.30, 2.40)
	**≥20%** (5, 29) vs. **<20%** (12, 71)	20.0% vs. 8.3% 0.508 2.75 (0.14, 55.17)	2.1 (1.2, 4.1) vs. 2.0 (1.6, 7.1) 0.247 1.98 (0.62, 6.32)
PD-L1 Score (*n* = 16) Cut-off (*n*, %)	**IC ≥ 1%** (9, 56) vs. **IC < 1%** (7, 44)	11.1% vs. 14.3% 0.849 0.75 (0.04, 14.58)	3.1 (1.2, 7.1) vs. 2.0 (1.6, NE) 0.749 1.20 (0.40, 3.62)
	**CPS ≥ 1** (9, 56) vs. **CPS < 1** (7, 44)	11.1% vs. 14.3% 0.849 0.75 (0.04, 14.58)	2.1 (1.2, 4.1) vs. 4.1 (1.6, NE) 0.181 2.28 (0.68, 7.63)
	**CPS ≥ 5** (9, 56) vs. **CPS < 5** (7, 44)	11.1% vs. 14.3% 0.849 0.75 (0.04, 14.58)	2.1 (1.2, 4.1) vs. 4.1 (1.6, NE) 0.181 2.28 (0.68, 7.63)
	**CPS ≥ 10** (7, 44) vs. **CPS < 10** (9, 56)	14.3% vs. 11.1% 0.849 1.33 (0.07, 25.91)	2.1 (1.2, 4.1) vs. 2.5 (1.5, 7.8) 0.402 1.60 (0.53, 4.82)
	**CPS ≥ 20** (6, 38) vs. **CPS < 20** (10, 62)	16.7% vs. 10.0% 0.699 1.80 (0.09, 35.42)	2.1 (1.2, 4.1) vs. 3.1 (1.5, 7.8) 0.150 2.41 (0.73, 7.98)
Combined TILs and PD-L1 (CPS) (*n* = 17) Cut-off (*n*, %)	**TILs ≥ 20% and CPS ≥ 10** (8, 24) vs. **TILs < 20% and/or CPS < 10** (9, 76)	12.5% vs. 11.1% 0.929 0.87 (0.05, 16.74)	2.0 (1.5, 7.8) vs. 2.1 (1.2, 4.1) 0.305 1.80 (0.59, 5.50)
	**TILs < 5% and CPS < 10** (3, 18) vs. **TILs ≥ 5% and/or CPS ≥ 10** (14, 82)	0% vs. 14.3% 0.968 NE	2.0 (1.9, NE) vs. 2.1 (1.5, 4.1) 0.360 2.03 (0.45, 9.21)

Abbreviations: TILs = tumor-infiltrating lymphocytes; CPS = combined positive score; IC = immune cell score; ORR = objective response rate; PFS = progression-free survival; CI = confidence interval; NE = not estimable.

**Table 4:** MDSCs levels (cells/µL) according to tumor response.

Time of Sample Collection	MDSC											
	Total				M-MDSC				G-MDSC			
	CB (SD ≥24 Weeks) (*n* = 4)		PD (*n* = 9)		CB (SD ≥24 Weeks) (*n* = 4)		PD (*n* = 9)		CB (SD ≥24 Weeks) (*n* = 4)		PD (*n* = 9)	
	Median	IR	Median	IR	Median	IR	Median	IR	Median	IR	Median	IR
Baseline	39.7 (*n* = 4)	37.7	55.7 (*n* = 8)	105.6	32.5 (*n* = 4)	20.4	47.7 (*n* = 8)	81.4	4.7 (*n* = 4)	25.0	9.0 (*n* = 8)	35.31
C3	12.9 (*n* = 4)	20.5	60.7 (*n* = 9)	243.7	9.8 (*n* = 4)	17.8	47.4 (*n* = 9)	223.1	3.5 (*n* = 4)	7.2	7.7 (*n* = 9)	23.4
C6/EOT	15.3 (*n* = 4)	15.4	37.9 (*n* = 4)	69.7	8.4 (*n* = 4)	18.2	21.5 (*n* = 4)	63.9	3.7 (*n* = 4)	7.0	11.8 (*n* = 4)	5.8
Baseline vs. C3 *p* value	0.0547		0.8438		0.0977		0.7422		0.1647		0.9453	
Baseline vs. C6/EOT *p*-value	0.1094		1		0.1484		1		0.1484		1	
Baseline CB vs. PD *p*-value	0.3502				0.2696				0.3502			
C3 CB vs. PD *p*-value	0.1897				0.2472				0.2472			
C6/EOT CB vs. PD *p*-value	0.1939				0.4705				0.1124			

Abbreviations: MDSCs = myeloid-derived suppressor cells; M-MDSCs = monocytic MDSCs; G-MDSCs = granulocytic MDSCs; CB = clinical benefit; PD = progressive disease; IR = interquartile range; Baseline: pretreatment plasma sample; C3: plasma sample collected at cycle 3; C6/EOT: plasma sample collected at cycle 6 and/or the end of treatment.

## Data Availability

The datasets collected and analyzed in our study are available from the corresponding author upon reasonable request.

## Supplementary Materials

The following are available online at https://www.mdpi.com/article/10.3390/cancers13215432/s1, Figure S1: Run-in phase design and patients inclusion, Figure S2: TILs density distribution according to OR (Yes/No), defined as CR + PR, Figure S3: PD-L1 IC (cut-off ≥1) distribution according to OR (Yes/No), Figure S4: TILs and PD-L1 levels correlation.
