# Supplementary material for: Pembrolizumab Plus Gemcitabine in the Subset of Triple-Negative Advanced Breast Cancer Patients in the GEICAM/2015-04 (PANGEA-Breast) Study
Source: Cancers (Basel). 2021 Oct 29;13(21):5432. doi: 10.3390/cancers13215432 (PMC8582406; doi:10.3390/cancers13215432)
Supplement: Supplementary file 1 [file cancers-13-05432-s001.zip › cancers-1417662-supplementary.pdf]

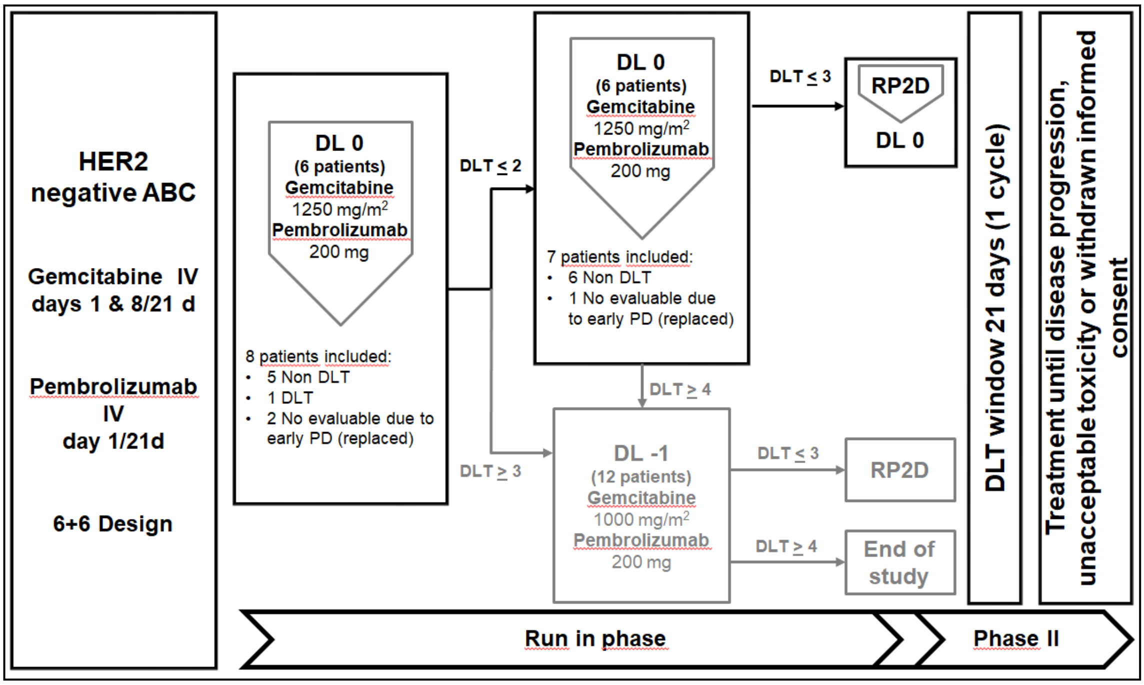

Figure S1. Run-in phase design and patients inclusion.

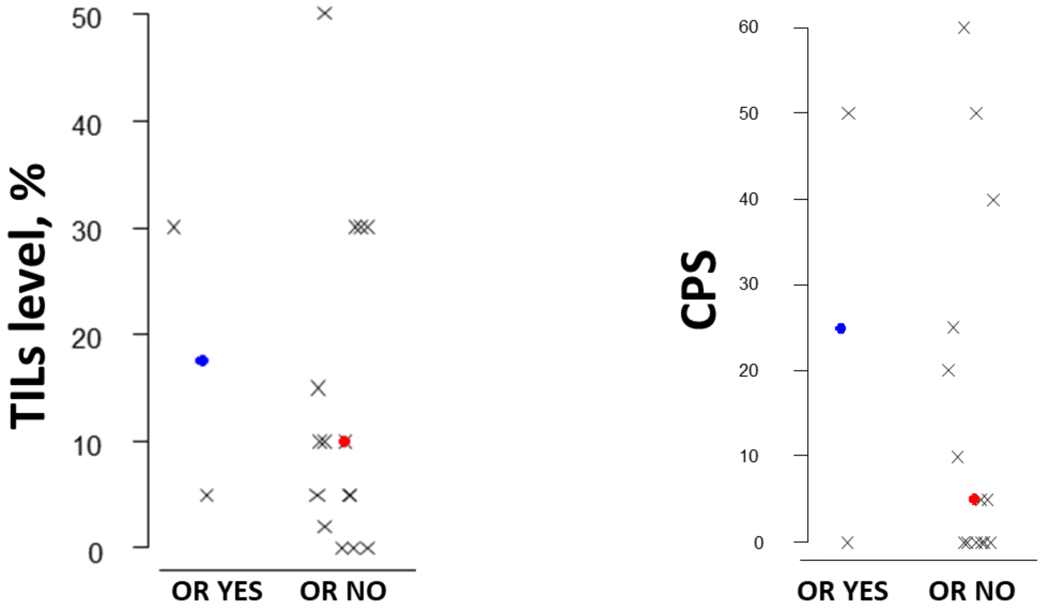

Figure S2. (**A**) TILs density distribution according to OR (Yes/No), defined as CR + PR. Blue and red dots represent TILs median values (%) in OR-Yes and OR-No subgroups, respectively. TILs % median: OR-Yes group = 17.5 (lower/upper quartiles 11.25/23.75) and OR-No group = 10 (lower/upper quartiles 3.5/22.5); Test Mann-Whitney p-value = 0.7045. (**B**) Median PD-L1 CPS distribution according to OR (Yes/No). Blue and red dots represent median values in OR-Yes and OR-No subgroups, respectively; CPS median levels: OR- Yes group = 25 (lower/upper quartiles 12.5/37.5) and OR-No group = 5 (lower/upper quartiles 0/23.75), Test Mann-Whitney p-value = 0.8681. TILs: tumour infiltrating lymphocytes; OR: Objective Response; TN: triple-negative; CPS: Combined Positive Score.

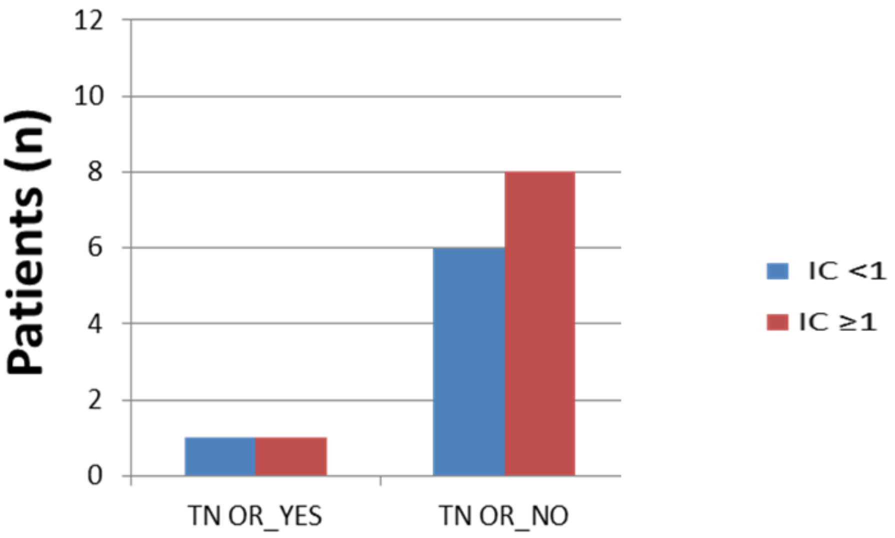

Figure S3. PD-L1 IC (cut-off ≥1) distribution according to OR (Yes/No).

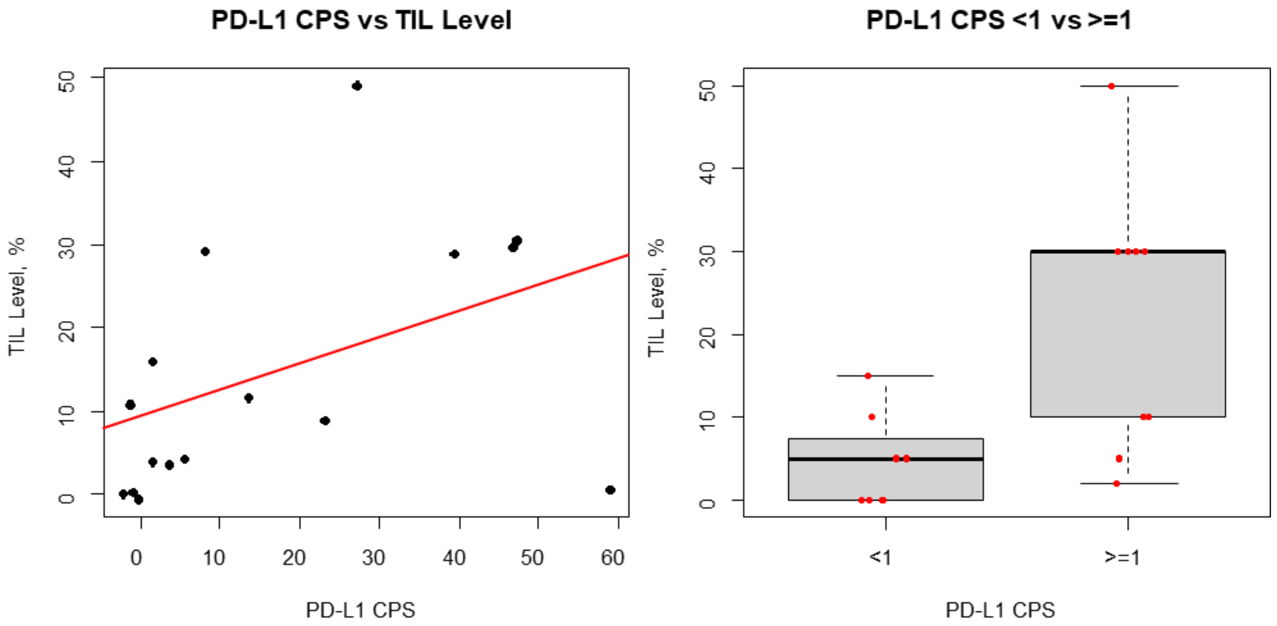

Figure S4. TILs and PD-L1 levels correlation. Spearman correlation coefficient = 0.5431836 (p-value = 0.02967). Mann-Whitney correlation p-value = 0.02384.
